# United Voices Group-Singing Intervention to Address Loneliness and Social Isolation Among Older People With HIV During the COVID-19 Pandemic: Intervention Adaption Study

**DOI:** 10.2196/60387

**Published:** 2024-10-08

**Authors:** Miranda Hill, Meredith Greene, Julene K Johnson, Judy Y Tan

**Affiliations:** 1 University of California, San Francisco San Francisco, CA United States; 2 Indiana University Indianapolis, IN United States; 3 Cancer Research Center for Health Equity, Division of Population Sciences Department of Biomedical Sciences Cedars-Sinai Medical Center Los Angeles, CA United States

**Keywords:** HIV, AIDS, mental health, loneliness, older adults, music-based interventions, technology, mobile phone

## Abstract

**Background:**

People living with HIV experience HIV stigma alongside a spectrum of aging-related health conditions that accelerate their vulnerability to the ill effects of loneliness and social isolation. Group-singing interventions are efficacious in improving psychosocial well-being among older people in the general population; however, the social curative effects of group singing have not been explored in relation to HIV stigma. By promoting group identification, bonding, and pride, group singing may reduce loneliness, social isolation, and other negative impacts of HIV stigma among older people living with HIV. Access to group-singing programs may be enhanced by technology.

**Objective:**

While group singing has been extensively studied in older adults, group-singing interventions have not been adapted for older people living with HIV to target loneliness and social isolation in the context of HIV stigma. The objective of this study was to describe the systematic development of a group-singing intervention to reduce loneliness and social isolation among older people living with HIV.

**Methods:**

In the San Francisco Bay Area between February 2019 and October 2019, we engaged older people living with HIV in a rigorous, 8-stage, community-engaged intervention adaptation process using the Assessment, Decision, Adaptation, Production, Topical Experts, Integration, Training, and Testing (ADAPT-ITT) framework. On the basis of a formative assessment of the needs and preferences of older people living with HIV, we selected an evidence-based group-singing intervention for older adults and systematically adapted the intervention components by administering them to a community advisory council (n=13).

**Results:**

The result was United Voices, a 12-week hybrid (web-based and in-person) group-singing intervention for older people living with HIV. United Voices comprises 12 web-based (ie, via Zoom [Zoom Video Communications]) rehearsals, web-based and in-person drop-in helpdesk sessions, and a professionally produced final concert recording.

**Conclusions:**

Through an iterative process and in consultation with stakeholders and topic experts, we refined and manualized United Voices and finalized the design of a pilot randomized controlled trial to evaluate the feasibility and acceptability of the intervention protocol and procedures. The findings provide insights into the barriers and facilitators involved in culturally tailoring interventions for older people living with HIV, implementing intervention adaptations within web-based environments, and the promise of developing hybrid music-based interventions for older adults with HIV.

## Introduction

### Background

Due to improvements in HIV treatment, people living with HIV are now commonly living into middle and older age. Antiretroviral therapy has resulted in better HIV treatment tolerability and sustained immunologic and virologic responses [1]. By 2030, approximately 73% of people living with HIV in the United States will be aged >50 years [2]. However, despite living longer, older people living with HIV are experiencing health challenges that include multimorbidity, polypharmacy, and premature onset of aging syndromes (eg, cognitive impairment and frailty) [3-6]. They are also susceptible to loneliness and social isolation [7]—2 conceptually distinct but related psychosocial outcomes independently linked to poorer mental health, quality of life, health behaviors (eg, smoking), and biological risk factors (eg, inflammation) [8-10]. In a meta-analysis of 70 prospective studies within the general population on mortality, loneliness (ie, feeling alone and socially disconnected and not belonging), and social isolation (ie, pervasive lack of social contact and participation in social activities), loneliness and social isolation were associated with a 14% and 32% increased likelihood of death, respectively [11].

Available evidence among older people living with HIV demonstrates that loneliness and social isolation are intricately linked to HIV stigma. Stigma is defined as an attribute that is socially discredited so that the person bearing the attribute (eg, HIV) is considered tainted, disgraced, or devalued [12,13]. People living with HIV remain highly stigmatized and are at risk of social isolation due to shrinking social networks (eg, friends passing away) and pre-emptive self-distancing to avoid potential rejection [14-16]. In a study with older people living with HIV, HIV stigma was found to be associated with loneliness, depression, and substance use and was a predictor of poor immunological response to HIV treatment [17]. HIV stigma contributes to social isolation among older people living with HIV through feelings of shame associated with having a disease that they are “too old to have” [18]. Despite the urgency of developing interventions that address the interconnected psychosocial needs of this growing and underserved population, there is a paucity of evidence-based interventions that specifically address the aforementioned factors [19].

By fostering improved social connections with peers, group singing is a promising intervention to counteract the negative effects of HIV stigma on social isolation and loneliness among older people living with HIV. Group singing is popular, easily interwoven into social and cultural traditions, and significantly linked to decreased loneliness and increased psychosocial well-being in older adults [20-22]. Furthermore, group singing is a low-cost activity requiring no previous skill or experience. Group singing improves psychosocial well-being via improved emotional well-being and increased feelings of belonging and social bonding [23-25], although evidence regarding how group singing may reduce loneliness and social isolation among stigmatized older persons remains scarce [26,27]. A meta-analysis found that, among the 50 behavioral interventions to reduce loneliness among older adults, none incorporated music or singing components, and only 1 targeted older people living with HIV (eg, a 12-week telephone group discussion–based intervention facilitated by cognitive behavioral therapists) and was not found to be efficacious [28]. Thus, the development of an adaptable, scalable, low-cost group-singing intervention holds the potential for improving psychosocial health among older people living with HIV.

### Study Purpose

We used the Assessment, Decision, Adaptation, Production, Topical Experts, Integration, Training, and Testing (ADAPT-ITT) framework to guide community-engaged research in developing a group-singing intervention for older people with HIV. ADAPT-ITT is a framework that guides the development or adaptation of evidence-based interventions to the needs, preferences, and attributes of populations over 8 sequential stages. Each stage in ADAPT-ITT builds upon the former to facilitate a flexible and iterative process for engaging community members in research activities that result in the development of a culturally congruent intervention. While initially developed to guide cultural adaptations of HIV behavioral interventions [29], this framework has been widely used to guide the adaptation of evidence-based interventions for new populations and new delivery settings [30,31].

We began our adaptation work focused on tailoring an evidence-based group-singing intervention for older adult psychosocial health, Community of Voices [21], to a new population—older people living with HIV—in 2020, at the height of the COVID-19 pandemic [32]. The pandemic required a pivot to a web-based setting and modality for all aspects of intervention delivery. With the emergence of the COVID-19 pandemic during this research, we also contribute knowledge and perspectives to support the development of social technology interventions for older people living with HIV and other populations with unmet psychosocial health needs. Social technology is defined as the use of technology mediums (eg, web-based video calls, chatting, and instant messaging) to enhance social connection, which may further reduce the loneliness felt by older people living with HIV [33]. We anticipate the findings to inform broader principles of behavior change to guide the development of interventions to mitigate the impact of stigma on psychosocial health among other stigmatized populations [32].

## Methods

In the following sections, we used the music-based intervention guidelines to report on the process of adapting Community of Voices using ADAPT-ITT [32]. Our music-based intervention checklist can be found in Multimedia Appendix 1.

### Assessment (Stage 1)

For the assessment, we recruited HIV care providers and people living with HIV from clinical and community-based settings for 2 separate qualitative research studies. In the first study, we conducted focus groups with HIV care providers and older people living with HIV at an HIV and geriatrics clinic to illuminate health issues and intervention needs among older people living with HIV [34]. In another study, we conducted semistructured interviews with 12 people living with HIV aged ≥50 years about their experiences of aging with HIV [35].

### Decision (Stage 2) to Testing (Stage 8)

We recruited and convened a community advisory council (CAC) of community stakeholders and advocates (n=13) in the HIV or AIDS communities locally as well as experienced musicians and choral directors of local community choirs. We recruited CAC members via our established community relationships as well as through the University of California, San Francisco, Center for AIDS Prevention Studies Community Engagement Core. The principal investigator initiated contact with potentially eligible individuals to describe the goals of the study and the role of the CAC and obtained informed consent from interested individuals. The 13 CAC members comprised 10 (77%) men and 3 (23%) women, of whom 12 (92%) were African American or Black individuals and 1 (8%) was a White individual, and their ages ranged between 50 and 69 years. The CAC convened 4 times over the course of 4 months via videoconference. Each session (75-90 minutes) was audio recorded and transcribed for qualitative data analysis. Trained research staff observed and took notes during all sessions.

We used community-engaged research to implement the activities in each of the 8 stages of the ADAPT-ITT model (Table 1).

Stage 1 of the ADAPT-ITT model (Assessment) is characterized by formative research on the unique determinants of the health issue with the new target population. The questions guiding this stage of inquiry were as follows: What are the behavioral and social factors that shape psychosocial health and HIV outcomes among the population? What type of intervention do they prefer? What are intervention gaps that align with unmet population health needs? In stage 1, we assessed the needs and health determinants of the population, older people living with HIV, through formative research.

The main objective of the Decision phase (stage 2) is to select an intervention to be adapted and adopted by the target population. The results of the assessment, along with literature reviews and expert consultations, are used to drive decision-making about the potential fit of an intervention for the primary health outcome. Other questions that shape decision-making processes may include the following: what is the capacity of an agency to implement an intervention? How will the intervention be delivered? What resources are available to facilitate adaptation? What type of intervention will be adopted by the focal population? On the basis of the needs assessment, we selected an evidence-based intervention to be adapted for the population, health issue, and psychosocial change mechanisms.

The primary goal of the Administration phase (stage 3) is to collect feedback on intervention components and necessary modifications to facilitate adaptation, uptake, and effectiveness for the population and health condition of interest. Primary questions guiding the Administration phase included the following: what intervention components need to be included, excluded, or modified? How should the original intervention be adapted for the population of interest? We obtained feedback on the intervention’s core components from key stakeholders via a CAC convened for this study. Specifically, we commenced intervention development activities to explore adaptations needed to (1) assess and improve the cultural relevance of Community of Voices for older people living with HIV and (2) facilitate intervention delivery using social technology versus in-person choir rehearsal and performance. As mandates for physical distancing took effect due to COVID-19, the research team convened the first CAC session to brainstorm how to navigate the unforeseen challenges related to implementing an in-person group-singing intervention aimed to address loneliness and social isolation among immunocompromised older people. At each CAC session, we generated ideas for modifying existing components and developing new ones using ADAPT-ITT activities (eg, group discussions and pretesting). After each session, the research team met to review and discuss CAC feedback and notes, generating specific impressions for the cultural adaptation of Community of Voices intervention components. Finally, the research team presented and collected feedback on the Community of Voices components and proposed procedures for a web-based group singing program along with examples of recent web-based choir performances.

The goal of the Production phase (stage 4) is to develop an initial draft of the adapted intervention in consideration of the original intervention components and underlying behavioral theory and mechanisms of change, resources for intervention implementation, and the cultural adaptions needed to fit the population of interest and the context of intervention delivery. Questions driving our production included the following: how are the core elements and key characteristics of the original intervention to be reflected and retained in the new intervention? What content and materials will be included in the new intervention? How will adaptations be documented? On the basis of findings from the CAC, we produced drafts of tailored intervention materials during this phase.

**Table 1:** Stages, project phase, and outcomes of the Assessment, Decision, Adaptation, Production, Topical Experts, Integration, Training, and Testing (ADAPT-ITT) framework for developing the United Voices intervention for older people living with HIV^a^.

ADAPT-ITT stage and goals	Project phase and research activities	Outcomes of study activities
Assessment (collecting information on population needs and the appropriate adaptation)	Conducted literature review on psychosocial health determinants among older people living with HIV Conducted in-depth interviews and focus group discussions with HIV care providers and older people living with HIV Reviewed evidence on psychosocial health interventions among older people living with HIV Published formative research	Identified loneliness and social isolation as unmet needs among older people living with HIV and desire for low-cost social network–based interventions that foster improved psychosocial well-being through connection and belonging Identified HIV stigma as a barrier to psychosocial health and well-being among older people living with HIV Identified knowledge and implementation gap in psychosocial interventions for older people living with HIV
Decision (selecting the appropriate intervention for adapting to a new population)	Conducted literature review on psychosocial health determinants among older people living with HIV Conducted in-depth interviews and focus group discussions with HIV care providers and older people living with HIV Reviewed evidence on psychosocial health interventions among older people living with HIV Published formative research	Identified group-singing intervention as appropriate given the needs of older people living with HIV Selected CoV^b^, an evidence-based group-singing intervention for older adults without HIV
Administration (obtaining feedback on the selected intervention and core elements)	Recruited CAC^c^ (n=13) of older people living with HIV and key stakeholders (ie, leaders in community choirs, HIV or AIDS survivorship advocacy, and faith-based organizations) Convened the CAC to elicit feedback on CoV and identify core elements and adaptation needs	Obtained CAC feedback on literature review, qualitative findings, and CoV intervention components Discussed the proposed intervention content, delivery strategies, and approaches based on their acceptability, feasibility, and relative importance Identified adaptations for cultural relevance, intervention components, and delivery for immunocompromised individuals during the COVID-19 pandemic
Production (producing drafts of intervention materials)	Analyzed CAC transcripts for intervention content development Recruited music team (2 music directors and 2 music producers) Iterated drafts of materials and core elements of the UV^d^ intervention for feedback from the CAC and topic experts	Established music team (2 music directors and 2 music producers) Selected music repertoire Identified music production needs Created first drafts of UV intervention manual, curriculum materials (eg, syllabus and study website), and protocols and procedures Drafted study questionnaire
Topic experts (consulting experts to obtain feedback on drafted materials and core elements)	Consulted CAC Consulted respective experts on HIV geriatrics and music-based intervention development	Elicited feedback on UV intervention manual, materials, protocols and procedures, and questionnaire
Integration (revising and finalizing intervention drafts based on previous feedback)	Music and research teams met weekly to integrate feedback from experts and CAC to refine core elements and synthesize intervention activities	Finalized syllabus, including timeline or schedule, Zoom (Zoom Video Communications) link, repertoire, and instructions for self-recording and uploading tracks Finalized study website Finished recording music directors’ tracks Finalized study procedures, including recruitment, eligibility, and data collection processes
Training (clarifying roles and training team members on intervention and study implementation)	Music and research teams met weekly to integrate feedback from experts and CAC to refine core elements and synthesize intervention activities	Established roles on research and music teams Trained staff on informed consent procedures, eligibility criteria, and data collection processes Finalized study implementation timeline for picnic meet and greet and “soft launch” events
(Pre-)testing (piloting components of the study procedures and protocol)	Convened in-person picnic for meet and greet Convened web-based soft launch before the first intervention session (ie, rehearsal)	Revised and finalized UV intervention manual and study procedures and protocols for pilot waitlist randomized controlled trial to evaluate the feasibility and acceptability of an adapted group-singing intervention for older people living with HIV

^a^ADAPT-ITT stages 1 and 2 occurred before the COVID-19 pandemic, whereas stages 3 to 8 occurred during the COVID-19 pandemic.

^b^CoV: Community of Voices.

^c^CAC: community advisory council.

^d^UV: United Voices.

The next 2 phases of ADAPT-ITT consisted of expert consultations with key stakeholders and the integration of that expertise into intervention development. We refined materials and drafted procedures by consulting topic experts (Topic Experts, stage 5), which we integrated into intervention drafts (Integration, stage 6). After developing the first draft of the intervention, we engaged in an iterative process of collecting feedback and expertise on the content and operational aspects of the intervention and integrating that feedback into successive intervention manual drafts. Questions such as the following drove the selection of topic experts and consultations: What expertise is needed to inform decision-making about intervention modifications outlined in the manual draft? What technology and concerns are needed to deliver the intervention online? What additional factors should be considered to increase the cultural relevancy of the intervention to the population within a given context? The insights emerging from the consultations were then integrated into new intervention plans and manual drafts during the Integration phase, which was led by the primary question of what changes needed to be made to improve the proposed intervention plans.

During the Training phase (stage 7), staff who are involved in intervention delivery undergo training led by a question of what protocols, skills, and resources are needed to prepare personnel for implementation. The final phase of ADAPT-ITT, Testing, is led by a prevailing question of whether the adaptations work. These questions are answered through a pilot test of the intervention with a small group of people followed by a randomized controlled trial examining the feasibility and acceptability of the intervention. We finalized roles and conducted necessary trainings on study trial implementation and intervention facilitation, music conducting, and music production.

We designed a pilot randomized waitlist-controlled study to evaluate the feasibility and acceptability of the intervention protocol and procedures with older people living with HIV.

### Ethical Considerations

All research participants provided verbal informed consent. Research activities were approved by the institutional review board at the University of California, San Francisco (study 21-34750). All research was performed in accordance with the regulations and guidelines set forth by the Declaration of Helsinki. Data were deidentified for analyses (ie, stripped of identifying information and identified using a unique number). Research participants received US $60 each for their participation in a CAC session.

## Results

### Assessment (Stage 1): Defining the Psychosocial Health Needs of Older People Living With HIV

Data from 2 focus groups with older people living with HIV and 2 focus groups with their health care providers identified unmet psychosocial health needs among older people living with HIV, including (1) pervasive feelings of loneliness and social isolation; (2) a strong desire for older people living with HIV–only spaces; and (3) a need for low-cost, group-based activities [34]. Specifically, in focus groups with older people living with HIV, participants expressed a need to participate in regular social activities with peers who share similar experiences [34].

In semistructured interviews with older people living with HIV concerning their experiences with aging with HIV and intervention needs, we found additional evidence to suggest (1) a desire for group-based interventions to counter the effects of social isolation due to HIV stigma and long-term survivorship and (2) a willingness to use digital technology to foster the development of peer support networks [35]. Another finding concerned the development of group pride and personal control through survivorship of the acute HIV and AIDS epidemic [35]. Participants described how they developed a positive perspective of aging with HIV in the years following HIV diagnosis [35]. Altogether, these formative assessment data laid the groundwork for deciding on an evidence-based intervention to be adapted for older people living with HIV.

### Decision (Stage 2): Deciding on an Intervention to Address the Psychosocial Health Needs of Older People Living With HIV

On the basis of the needs identified in stage 1 (Assessment), the research team (ie, the principal investigator [JYT] and 2 research assistants) reviewed the literature to identify effective and cost-effective group-based interventions focused on promoting psychosocial well-being through increased social support and decreased loneliness. On the basis of our review of this literature, we consulted with experts on music-based interventions in older adults (JJ) and HIV geriatrics (MG). The formative assessment data, literature, and expert consultations led us to select the Community of Voices intervention, an evidence-based community group-singing intervention for racially or ethnically and socioeconomically diverse older community-dwelling adults [21]. Community of Voices was designed to promote cognitive, social, and physical engagement through weekly 90-minute sessions in which participants took part in vocal exercises, singing, discussions, socialization, and quarterly public performances. In a 12-site, randomized waitlist-controlled trial, the intention-to-treat comparison at 6 months showed that, compared to control participants, intervention group participants experienced significantly greater improvements in loneliness (*P*=.02; standardized effect size=0.34) and interest in life (*P*=.008; standardized effect size=0.39) [21]. Community of Voices was selected due to its positive impacts on loneliness and social isolation, the psychosocial outcomes of interest in this study, among racially, ethnically, and socioeconomically diverse older adults. In addition, group singing was selected over other activities such as painting or pottery making, which are more independent activities, in favor of its emphasis on cocreating music, a group-based, interdependent activity that can promote social bonding and group pride. Finally, Community of Voices was cost-effective, with minimal barriers to entry for those inexperienced in singing. Altogether, there was evidence suggesting a potential for achieving similarly positive outcomes when tailored for older people living with HIV.

### Administration (Stage 3): Sharing and Receiving Feedback on the Intervention From Community Stakeholders

#### Intervention Components

In the first CAC session, members were introduced to the research goals and the current state of knowledge on older adult psychosocial health factors (including loneliness, social isolation, and aging syndromes in older people living with HIV). The CAC was strongly supportive of the Community of Voices component focused on psychosocial engagement to improve emotional well-being, social support, loneliness, and interest in daily life through exposure to the uplifting effects of singing, building a social network, and having something interesting to do [36]. They also supported the inclusion of the cognitive component, which focused on improving memory, verbal learning, and mental stimulation through learning and recalling music and attending to the conductor. The physical engagement component of the intervention, which aimed to promote physical health through standing, sitting, and moving to the music and around the room while socializing, was not determined to be feasible to implement given COVID-19 risks and restrictions on in-person gatherings.

#### Cultural Relevance

The CAC discussed the adaptations necessary for improving the cultural relevance of the intervention, and discussions centered on the intervention content and the group’s social identity. One CAC member who was a key stakeholder in the community suggested that the singing group be called United Voices, and all members of the CAC agreed. Two key issues on the social identity of United Voices were discussed: (1) HIV and anti-LGBT+ (lesbian, gay, bisexual, transgender, and queer) stigma and (2) the explicit identity of United Voices as a group for people living with HIV (vs being HIV status neutral). On the issue of stigma experienced by people living with HIV related to HIV and being members of the LGBT+ community, CAC members discussed mixed experiences of HIV stigma and homophobia in churches, particularly in predominantly Black or African American churches. One CAC member expressed the need for the singing group to be inclusive of people who are atheist and agnostic and, additionally, the need to ensure that the intervention was spiritual in nature as opposed to being gospel oriented—which was consistent with the original Community of Voices intervention protocol but emphasized by the CAC when discussing the intervention within the context of HIV stigma. One participant stated the following:

That’s the thing I would like to focus on...because I, I don’t want to offend anybody here, but I’m an atheist. I don’t belong to a church...I know a lot of people in my situation, too...I’m not sure about words for it. But for me, inclusion means the ability to worship at your own pew or no pew at all.

Another stated the following:

I’m just saying the church has boxed everybody in and gave everybody this stigma and this guilt feeling...We need to reach outside the world—outside the walls and make people, uh, feel loved.

Discussion also emerged regarding whether it was necessary to identify the singing group as a group for those living with HIV or simply label the group as “HIV status neutral.” The advantages of creating an HIV status neutral singing group included ease of participant recruitment from not having to screen for and verify HIV serostatus. Some CAC members also felt that it would perhaps remove potential barriers to participation by people living with HIV who may not wish to disclose their HIV status by participating in a group designated for people living with HIV. On the other hand, members of the CAC and research team strongly felt that, to create a sense of pride and reclaim a “spoiled identity” around HIV and aging, it was necessary to explicitly label United Voices as a singing group specifically for older people living with HIV. Discussions among the research team, topic experts, and CAC members led to a joint decision to implement the research as designed to allow for testing of the mechanisms of action proposed but to ask participants in exit interviews about the acceptability of the group being HIV status neutral and potential drawbacks and advantages to inform future research trial design.

Part of the conversation surrounding the cultural relevance of the intervention through web-based methods centered on how to replicate and tailor the core ingredients focused on bonding and group pride. As opposed to having people bond through physical singing and conversation, as was done in the Community of Voices intervention, members of the CAC expressed that bonding and group pride would be facilitated through careful selection of the songs for the adapted intervention. Specifically, they emphasized that unity could be promoted through discussions of the context and meaning of the songs among the intervention participants.

Two web videos of choir performances were shown during a CAC meeting, one from the San Francisco Gay Men’s Chorus [37] and another from Eric Whitacre’s Virtual Choir [38]. CAC members expressed feeling moved by the web-based choir performances that were shown during the first session that illustrated how web-based group singing may be achieved. Responses from CAC members included the following:

...*it baffles me that anybody can put two people in a room and make them sing together, but that many people singing in harmony at the same time in separate places blows my mind. But I was very touched and very moved by it, and I thought it was a brilliant example of the power of it*.

I thought it was very, very well done, um, very emotional in terms of, you know, the impact that it brings ’cause they’re all singing together but singing separately. And it’s amazing that that software is, is present and available for us all to use and utilize. And, and, uh, it’s, it’s a beautiful thing.

While some expressed some concerns about the technical aspects of the technology-driven process, they highlighted the potential of reaching a larger target audience through web-based intervention delivery with the help of technological experts and those experienced with administering web-based choir performances. Other modifications that were suggested by the CAC included reducing the number of intervention sessions (ie, rehearsals). They also suggested that tutorials on how to use the technology would facilitate technological literacy, consequently encouraging intervention adoption and uptake by the participants.

### Production (Stage 4): Manualizing the Intervention and Materials for United Voices

In total, 4 musicians (n=2, 50% music directors and n=2, 50% producers) comprised the music team to work with the research team to adapt the intervention content from stage 4 and implement United Voices. The music and research teams met 1 to 3 times a week over a period of 2 months and selected the music repertoire; identified production needs; and drafted a syllabus containing detailed information on rehearsal times and Zoom (Zoom Video Communications) links, contact information (email and phone) and biographies of the music and research team members, study assessment timeline (see stage 8, Pretesting, for trial design), ground rules and expectations for singers, weekly rehearsal schedule, and instructions on creating audio and video files for the final web-based concert. The rehearsal schedule was developed to include sufficient time for learning and rehearsing each new song, consulting with and learning from the music team on technicalities of audio and video recording at home. Instructions on how to prepare audio and video recordings were drafted by the music producers and were written for a lay audience with low technology literacy and minimal requirements for devices and equipment. Figures and step-by-step instructions were drafted and included in the syllabus (Multimedia Appendix 2). Meetings involved an open discussion of proposed intervention content, delivery strategies, and approaches based on their acceptability, feasibility, relative importance, and perceived value. The research team synthesized and organized these data to draft the intervention manual.

A website housed information pertinent to the study that included general study information and introduction, brief biographies of the research and music team members, lyrics to songs, music tracks recorded by each music director for singers to listen and sing to while rehearsing on their own, and the syllabus and additional instructions on how to upload recordings.

### Topic Experts (Stage 5) and Integration (Stage 6): Gathering and Integrating Feedback From Field and Community Experts

The next 2 stages of ADAPT-ITT comprised expert consultations with key stakeholders and the integration of that expertise into intervention development. After developing the first draft of the intervention, we engaged in an iterative process of collecting feedback and expertise on the content and operational aspects of the intervention and integrating that feedback into successive intervention manual drafts. Expert consultations consisted of CAC sessions and meetings with a subject expert on group-singing interventions (JJ) and HIV geriatrics medicine (MG). The CAC brainstormed names of potential music directors with experience working with older populations, conducting web-based group-singing rehearsals, and producing web-based concerts with recordings. In consultation with the topic expert and clinician, the principal investigator (JYT) interviewed candidates to ensure that they had the expertise, training, and experience regarding directing web-based singing groups with older adults who may not have had previous singing experience. It was especially important to the team that music directors and producers of United Voices had either lived experience or experience working with people with stigmatized identities (eg, being LGBT+ or living with HIV).

The feedback from experts fell into two domains: (1) technology-based considerations, logistics, and innovations and (2) modifications to improve the cultural relevance of the intervention for older people living with HIV. While there was some debate among the research team, the music team, and the CAC about rehearsal length and frequency given the web-based format of the intervention, prevailing feedback promoted the importance of having choir members meet weekly for 90 minutes (as done in the original intervention) to facilitate socialization, connection, and breakout rehearsals by voice parts using breakout rooms. Recommendations for supporting web-based engagement were integrated into a syllabus (Multimedia Appendix 2) that contained ground rules for participation, including an attendance policy with a maximum of 2 excused absences from choir rehearsals. As in the original intervention, music directors were to also prompt reflection on the songs’ meanings and relevance. Suggested modifications to promote the cultural relevance of this intervention for older people living with HIV focused on having music directors select songs with themes that were uplifting and encouraging and could be used to evoke communal reflection and dialogue about the messages within the context of life experiences. To that end, this adaptation was to be implemented by having music directors begin rehearsals with a discussion of the song’s themes, messages, and why they selected the song for the choir. It was also determined that, given their expertise and leadership of the rehearsals and performance, music directors would play a central role in keeping participants interested and engaged by actively soliciting input from other participants during rehearsals and establishing a rapport with them through weekly office hours.

In response to concerns about building efficacy and confidence in learning music while using technology to engage in the singing component, several modifications were made to the protocol. These modifications included assessing participants’ technological literacy and resources before the intervention (and offering personal and financial assistance to those who did not have the skills or resources), having them preview the song repertoire in the syllabus during the orientation session, listening to reference audio tracks that were sent via email, and using 2 mobile devices for listening along to the track while also recording their music part. Participants were also to send their audio recordings to producers as soon as possible to allow time for the producers to combine the videos and sounds into a cohesive chorus. Other recommendations from topic experts that led to cultural and technological innovations are detailed in Multimedia Appendix 3.

### Training (Stage 7): Establishing and Clarifying Roles and Training Staff for Intervention Delivery

The research team discussed training for the formative pilot study during weekly meetings that began during expert consultations. During the training, the study goals, procedures, and manual were reviewed with a focus on detailing the stages for facilitating the community group-singing model outlined in the manual alongside a syllabus describing the intervention activities and operational logistics. For example, the project coordinator (HCK) received targeted training on how to manage, coordinate, and store data, whereas the music producers received training on Zoom features that would be used in the choir rehearsals, such as breakout rooms for socialization and coaching. The meetings also served as a forum for identifying information gaps and questions among staff.

### Testing (Stage 8): Examining How Well the Intervention Was Adapted Through Pilot Research

During the final stage, the researchers piloted the study protocols (including informed consent and the rehearsal model) and intervention through a rehearsal in which staff and CAC members role-played their respective roles, responsibilities, and social interactions using the technology platforms and tools that would be used in the study. Additional modifications were made to refine the intervention and assist with technology use through the development of detailed project instructions with diagrams and video links that provided visual and auditory support. Multimedia Appendix 2 shows the revised syllabus with diagrams that were added after the pilot.

A Zoom-based soft launch of the United Voices intervention was set for a date before the start of the intervention pilot, a randomized waitlist-controlled design. Participants randomized to the intervention arm were invited to the soft launch. The goal of the soft launch was to introduce to participants the goal of the research study, the research team members, the music producers, and directors.

A week before the first rehearsal, printouts of study materials (ie, syllabus and lyrics) were organized into a folder with plastic protective sheets and mailed to participants. The intervention featured uplifting up-tempo gospel song selections characterized by energetic rhythms; lively instrumentation; and joyful, powerful messages of faith and hope. The vocal arrangements featured strong, soulful voices delivering lyrics that spoke of overcoming obstacles, finding strength in faith, and celebrating the goodness of life. Harmonies were rich and layered, with the choir adding depth and power to the songs.

In addition, the study team held a 2-hour outdoor, masked, and catered community event in Oakland, California, on July 17, 2021. The meet and greet was designed to supplement the remote, web-based nature of the study and create a positive in-person experience and personal connections. This was for participants to meet other participants, the study team, and music directors or producers. The study team provided an overview of the study, and the music directors or producers introduced themselves to the participants. Refreshments were served. A total of 5 participants attended.

We finalized the design of a 12-week, pilot randomized waitlist-controlled trial of the United Voices intervention with older people living with HIV (N=23) to evaluate the acceptability and feasibility of implementation and assessment procedures and protocols, including randomization procedures. The results of that trial will be published separately.

## Discussion

### Principal Findings

In this paper, we describe the development of a web-based group-singing intervention, United Voices, for older people living with HIV. Our main findings suggest that group-singing interventions for older people living with HIV may be promising approaches to psychosocial health promotion via social technology.

In addition, our findings contribute novel information to guide the development of culturally relevant interventions for older people living with HIV that address HIV stigma, social isolation, and loneliness using social technology. In this section, we discuss key lessons learned from the study to facilitate future cultural and social technology intervention adaptations for older people living with HIV.

The cultural adaptations for United Voices were critical to the integrity of the core components, behavior change mechanisms, and desired outcomes among older people living with HIV. Adaptations were informed by the collective expertise of researchers, community members, and formative data, which altogether fostered the addition of necessary population- and health-specific constructs to the theoretical framework underlying the study. These adaptations began with the development of a new tailored framework that recognized the negative impacts of HIV stigma on psychosocial well-being among older people living with HIV through belongingness, group pride, and personal control [39,40]. In doing so, we designed an intervention that recognizes and seeks to intervene in the detrimental impacts of HIV stigma on social isolation through a strength-based approach (vs being deficit based). In a recent editorial, Poteat and Logie [41] stated that “a deficits perspective in HIV research can overlook the skills, knowledge, and collective resources within marginalized communities,” whereas strength-based research can locate and support existing health-promoting resources that can mitigate the impact of stigma, “identify the mechanisms of action underlying protective factors,” and “reveal unexamined assumptions and generate novel approaches.” To that end, our formative assessment yielded new information about how HIV stigma can foster individual and collective pride, personal control, and improved psychological well-being through long-term survivorship among older people living with HIV—a mechanism of action that has been overlooked within the deficit-based research that predominates HIV literature. In turn, the aforementioned revelation became the foundation for culturally tailoring the intervention, which was further refined to address and mitigate the negative impacts of stigma on psychosocial wellbeing by harnessing the health promotional physiological and social mechanisms of group-based singing and discussions centered on these topics.

Our study demonstrated that videoconferencing is a viable platform for implementing group-singing interventions with older people. The engagement, enthusiasm, and advice from experts and key informants within the CAC encouraged study continuation through a web-based forum despite potential barriers noted among older adults in the literature, such as lack of access to a technical device and lack of experience with technology [42]. Our ability to address such barriers through the provision of resources, technical assistance, tutorials, and diagrams is largely accredited to our probing into attitudes toward social technology interventions among older people living with HIV during our formative assessment, the advent of web-based group-singing interventions during the COVID-19 pandemic, and our continual inclusion of key community stakeholders throughout the intervention development process. The adaptation of Community of Voices into United Voices was systematically guided by the ADAPT-ITT framework, which includes the direct involvement of members of the population of interest, key stakeholders, and staff from the initial to last stage and “the use of topical experts to assist in creating the adapted intervention” [29]. The steep upward trend in technology use among older adults over the past decade may have also contributed to our success in developing a web-based singing intervention. Recent data on technology use among older adults show that the percentage of adults aged ≥65 years who own a smartphone increased by 50 percentage points from 2012 to 2021, with 61% of adults aged ≥65 years owning a smartphone in 2021 compared to 11% who owned a smartphone in 2012 [43]. Moreover, nearly half of older adults own a tablet or computer, and social media use was 4 times higher among older adults in 2021 than it was in 2010 [43]. Other research on socialization-driven technology use among older adults during the COVID-19 pandemic also suggests that a rise in social motivation and interest in using social technology to connect with others during the pandemic, along with the ease of participating in social activities that were facilitated by others on the web, may have been another contributing factor [44]. Despite the decline in COVID-19–related morbidity and mortality, our study data, along with these population trends, indicate many possibilities for growing the breadth of social technology interventions in HIV and other health areas for older adults. While social technology–based interventions for older adults may be less urgent given that COVID-19 is no longer a public health emergency, there is some evidence to suggest that such interventions can significantly improve mental and social health outcomes among older adults. One study that evaluated the effectiveness of remote evidence-based interventions (including web- and telephone-based delivery) among older adults living with a variety of chronic diseases found that interventions led to significant improvements in overall health, depressive symptoms, loneliness, and self-efficacy in technology use among participants [45]. Such interventions may be particularly important among those who have access barriers to participating in evidence-based interventions, such as those who live in geographic areas where transportation or travel presents potential barriers to intervention engagement. The findings of this study may be used to inform intervention designs with older people living with HIV to accommodate the realities of a post–COVID-19 world in which hybrid participation comprising a combination of in-person and remote activities is now possible and expected.

### Strengths and Limitations

The predominance of Black or African American individuals in the CAC is a strength given the disparately high burden of HIV and HIV- or AIDS-related stigma among Black people in the United States [46]. Access to mental health services is also significantly lower among Black or African American individuals even though they experience similar levels of serious psychological distress compared to other racial and ethnic groups [47]. Thus, although their perspectives on cultural tailoring likely align with those of a subgroup of older people living with HIV that is most in need of a group-singing intervention, we would likely have identified additional intervention needs with a more racially and gender-diverse advisory group. For example, cross-cultural experiences with churches and songs with more diverse groups may have shaped decision-making regarding the music repertoire. Our decision to integrate a discussion prompt about social isolation, stigma, religion, and music within the intervention sought to address this potential limitation by allowing music directors to flexibly navigate and tailor song selection in partnership with the choir and within the context of their lived experiences in the aforementioned areas.

While a large share of intervention development activities took place remotely and on the web, the assembly of the research team and recruitment of the CAC took place in person before the onset of the pandemic. Community outreach and recruitment were also conducted through the distribution of study advertisements on the web and in person. Thus, our implementation was not entirely web-based, and it is possible that having pre-established in-person interactions and community outreach is necessary to ensure the success of conducting activities on the web.

While we initiated intervention development before the COVID-19 pandemic, using ADAPT-ITT, an evidence-based community-engaged intervention adaptation framework, enabled implementation pivots that were necessary, innovative, and rooted in a community-driven process. Nevertheless, a potential limitation lies in our deviation from stage 8, the intervention testing stage of ADAPT-ITT, which involves conducting a pilot test (stage 2a) with “approximately 20 participants of the new target population” [29] of the final draft of the intervention manual before the stage2b randomized controlled trial. Limited resources and time due to COVID-19 pandemic delays did not allow for this stage to be fully implemented. Instead, we conducted stage 2a testing with the CAC. While this detour still permitted us to answer the question of how successful the evidence-based intervention adapted to the new population was, it is possible that the positive feedback from the CAC limited the collection of impartial insights that we would have obtained from participants who were naïve to the intervention.

The findings of this study resulted from an intentional and continuous engagement throughout the study of the older communities in the San Francisco Bay Area impacted by HIV and AIDS. Indeed, a strength of this study was its use of the ADAPT-ITT framework to guide the adaptation of Community of Voices into United Voices, consistent with a community-engaged research approach. Researchers who are interested in using our findings to develop similar interventions must include in their designs the intentional engagement of the ultimate end users of the intervention throughout the research life cycle.

### Conclusions

Relative to their HIV-negative counterparts, older people living with HIV are more likely to report loneliness and, thus, constitute a psychosocially vulnerable population. In this study, we used the ADAPT-ITT framework to guide and successfully transition and innovate intervention adaptation and delivery on the web during the COVID-19 pandemic in partnership with experts, community members, and key community stakeholders. Social technology–delivered group-singing interventions are possible and potentially powerful in extending the reach of intervention development and implementation. Future trials include the efficacy testing of a hybridized (ie, a combination of in-person and remote modalities) United Voices intervention with older and racially, ethnically, sexually, and gender-diverse communities impacted by HIV or AIDS.

## Appendix

Multimedia Appendix 1 Music-based intervention reporting criteria.

Multimedia Appendix 2 United Voices syllabus.

Multimedia Appendix 3 Topic expert recommendations and adaptations for COVID-19 and cultural relevance for older people living with HIV.

## Abbreviations

ADAPT-ITT: Assessment, Decision, Adaptation, Production, Topical Experts, Integration, Training, and Testing
CAC: community advisory council
LGBT+: lesbian, gay, bisexual, transgender, and queer

## Data Availability

The datasets generated during and analyzed during this study are available from the corresponding author on reasonable request.
