# Supplementary material for: United Voices Group-Singing Intervention to Address Loneliness and Social Isolation Among Older People With HIV During the COVID-19 Pandemic: Intervention Adaption Study
Source: JMIR Form Res. 2024 Oct 8;8:e60387. doi: 10.2196/60387 (PMC11496909; doi:10.2196/60387)
Supplement: Multimedia Appendix 1 [file formative_v8i1e60387_app1.pdf]

Music-based Intervention Reporting Criteria	Page:
**A: Intervention Theory**	
Provide a rationale for the music selected; specify how qualities and delivery of the music are expected to impact targeted outcomes.	13-18
**B: Intervention Content**	
Provide precise details of the music intervention and, when applicable, descriptions of procedures for tailoring interventions to individual participants.	
**B.1: Person Selecting the Music**	
Specify who selected the music: (1) pre-selected by investigator, (2) participant selected from limited set, (3) participant selected from own collection, or (4) tailored based on patient assessment.	13 - 15
**B.2: Music**	
When using published music, provide reference for sheet music or sound recording.	16
When using improvised or original music, describe the music's overall structure (i.e., form, elements, instruments, etc).	16
**B.3. Music Delivery Method (Live or Recorded)**	
When using live music, specify who delivered the music and the size of the performance group (e.g., interventionist only, interventionist and participant).	N/A
When using recorded music, specify placement of playback equipment and the use of headphones vs. speakers. Specify who determined/controlled volume (e.g., interventionist; participant. Specify decibel level of music delivered and/or use of volume controls to limit decibels.	15 – 17 & Appendix B
**B.4: Intervention Materials**	
Specify music and/or non-music materials.	15 & Appendix B
**B.5: Intervention Strategies**	
Describe music-based intervention strategies under investigation (examples: music listening, songwriting, improvisation, lyric analysis, rhythmic auditory stimulation, etc).	12, 15 - 16
**C: Intervention Delivery Schedule**	
Report number of sessions, session duration, and session frequency including practice sessions.	Appendix B
**D: Interventionist**	
Specify interventionist qualifications and credentials.	7 – 8, 14-16
Specify how many interventionists deliver study conditions.	7-8, 13
**E: Treatment Fidelity**	
Describe strategies used to ensure that treatment and/or control conditions were delivered as intended (e.g., interventionist training, manualized protocols, and intervention monitoring).	7-8, 13-14
**F: Setting**	
Describe where the intervention was delivered; include location, privacy level, and ambient sound.	13-17, 18
**G: Unit of Delivery**	

Specify whether interventions were delivered to individuals or groups of individuals, including the size of the group	7-8, 18
