# Supplementary material for: United Voices Group-Singing Intervention to Address Loneliness and Social Isolation Among Older People With HIV During the COVID-19 Pandemic: Intervention Adaption Study
Source: JMIR Form Res. 2024 Oct 8;8:e60387. doi: 10.2196/60387 (PMC11496909; doi:10.2196/60387)
Supplement: Multimedia Appendix 2 [file formative_v8i1e60387_app2.pdf]

SYLLABUS

Rehearsals

Wednesdays, 5 – 6:30 PM

August 25 – November 10, 2021

Zoom: [redacted]

Musical Team	Research Team
[redacted] Musical Co-Director<br>[redacted]	Phone: [redacted]<br>Email: Unitedvoices@ucsf.edu<br>Website: www.unitedvoices.ucsf.edu
[redacted] Musical Co-Director<br>[redacted]	Cindy Kim, Study Coordinator<br>[redacted]
[redacted] Music Production Coordinator<br>[redacted]	Jovon Bright, Recruiter<br>[redacted]
[redacted] Music Producer<br>[redacted]	Robert Williams, III, Community Liaison<br>[redacted]
	Dr. Judy Tan, Associate Professor of Medicine, Principal Investigator<br>[redacted]

WHAT IS THIS RESEARCH STUDY?
The *United Voices* study is a research study funded by the National Institutes of Health (NIH) and conducted by Dr. Judy Tan to explore how group singing may benefit mental health and well-being.

WHAT SHOULD I EXPECT?
As a choir member, you should expect to:

- **Learn!** Learn and record 8 songs throughout the 12 weeks and learn the history behind them.
- **Socialize!** Get to know your fellow choir members.
- **Sing!** You will be asked to upload a recording of yourself singing each song. The Music Production Team will assemble these recordings into a final virtual performance.
- **Attend every rehearsal!**
- **Be on-time**! Arrive at each rehearsal on time and remain until the rehearsal is over.
- **Complete 3 surveys and an exit interview**! As part of this research study, a survey will be emailed to you 3 times over the course of 12 weeks. You will also be invited for an exit interview with Dr. Tan.

WHAT WILL I NEED?

- Enthusiasm!
- 2 devices: one for recording yourself singing and the other to listen to the reference track. (If you do not have two devices, no problem. Let the Research and Music Team know.)

GROUNDS RULES AND EXPECTATIONS

1. Be present!
    a. Have camera on for the duration of the rehearsal.
    b. Avoid distractions.
2. Do your best to show enthusiasm and be alert.
3. Join rehearsals from quiet, private space where you can be by yourself.
    a. Mute yourself if there is too much background noise.
4. Maintain confidentiality – what is said in the (Zoom) room stays in the room.
5. "Don't yuck my yum" – don't put others down
6. Create an environment of respect and gratitude. Recognize everyone has different points of views, and it's okay for everyone to have different points of views.
7. Use 'Raise hand' feature if you'd like to speak while another person is speaking.

ATTENDANCE POLICY

The study only allows a certain number of people in each choir. Therefore, your absence will be noticeable and will take away from the group's experience. We understand that life happens.
Out of courtesy to others and your musical team, if you must be absent, we ask that you let Dr. Roberts or Wilson know ASAP. Two or more absences may terminate your participation.

BIOGRAPHY

Research Team

Judy Tan, PhD, MA (she/her)
Dr. Judy Y. Tan is a social psychologist and Associate Professor in the Division of Prevention Science, Center for AIDS Prevention Studies, at UCSF. Dr. Tan also trained in vocal music at the "Fame" School, LaGuardia High School of Music, Art, and Performing Arts in New York City. She is interested in how social inequality manifests in health disparities among racial/ethnic, sexual, and gender minority communities in the U.S. Dr. Tan is an immigrant and the first in her family to attend college. She identifies as queer, Asian/Pacific Islander, and cis woman.

Hyunjin Cindy Kim (she/her)
Hyunjin (Cindy) Kim has a background in Public Health, most specifically in Behavioral Science and Health Education. As a coordinator on another HIV research project, Cindy is excited to work with United Voices as she would like to work towards addressing health disparities and engage with communities living with HIV. She believes that there is more work to be done to eliminate stigma surrounding HIV.

Jovon Bright (he/him)
Jovon has experience with social work and clinical work serving people living with HIV, People of Color, transgender and same-gender loving clients. Since Jovon arrived in the bay area in 2008, Personal connection to the work has driven his engagement and development. He has volunteered, become a certified HIV/HepC Counselor, and achieved a Phlebotomy certification, becoming increasingly involved to HIV specialized work.

Robert Williams, III (he/him)
Robert Williams is a native of San Francisco and was raised in Oakland, CA which is where he still calls home, and where he remains closely connected to his family, friends and community. As a Black gay man, he has been working on the HIV prevention needs of men since 1993, but even before then he was personally impacted by HIV as he saw close friends pass from the devastation of this disease. The memory of those friends and seeing other friends still contracting HIV fosters his ongoing commitment to this work.

REHEARSAL SCHEDULE

Week #	Date	Weekly Activity, Wednesdays, 5 – 6:30 PM Pacific	Singer's Recording Uploaded
Soft Launch	*Aug. 18*	*Introductions. Review technical procedures*	
1	Aug. 25	- Learn *Here's the Reason*<br>- Complete Survey #1	
2	Sep. 1	- Tweak *Here's the Reason*<br>- Learn *Better*	
3	Sep. 8	- Learn *Better, You Are My Strength*	
4	Sep. 15	- Tweak *Better, You Are My Strength*	
5	Sep. 22	- Learn *I Really Love You* and MAYBE *There Must Be Love* (if enough time)	
6	Sep. 29	- Tweak *I Really Love You*<br>- Learn *Be Healed*<br>- Complete Survey #2	
	Fri, Oct. 1		
7	Oct. 6	- Tweak *Be Healed*<br>- Learn *There Must Be Love*<br>- Learn *I Need You to Survive*	
	Fri, Oct. 8		
	Tue, Oct. 12	Help Desk time with Rusty and Paul @ 5pm Pacific	
8	Oct. 13	- Tweak *Be Healed, There Must Be Love*<br>- Production begins	
	Thu, Oct. 14	Help Desk time with Rusty and Paul @ 5pm Pacific	
	Fri, Oct. 15		Send in recording for *You Are My Strength*
9	Oct. 20	Production	
	Thu, Oct. 21	Help Desk time with Rusty and Paul @ 5pm Pacific	
	Fri, Oct 22		Send in recording for *Better*
10	Oct. 27	Production	
	Thu, Oct. 28	Help Desk time with Rusty and Paul @ 5pm Pacific	
	Fri, Oct 29		Send in recording for *Be Healed*
11	Nov. 3	Production	
	Fri, Nov. 5		

12	Nov. 10	- Virtual Concert Recording<br>- Complete Survey #3	
	Fri, Nov. 12	-	Send in recording for *I Really Love You*
Joint Virtual "Concert"	*Sunday, Feb. 6 (Tentative)*	*Roberts, Wilson, Watson, & Daniels Present: THE UNITED VOICES JOINT CONCERT EVENT*	

IT IS CRITICAL THAT SINGERS SUBMIT AND UPLOAD THEIR RECORDINGS AS SOON AS POSSIBLE.

VIRTUAL CHOIR PROJECT INSTRUCTIONS

Hello Choir! Thank you for taking the time out to make some music together!
This handout will give you the steps and instruction to record your video as part of the virtual choir.
These instructions have been adapted by Paul Daniels from www.developlayllc.com/virtualchoir.

What you'll need:

- 2 devices
  - a smartphone (for recording)
  - a tablet/computer (for watching and listening)
- Earbuds or headphones (to use when listening to reference track)

It's easy as 1 - 2 - 3!

1. Rehearse the song
2. Record a video of you singing with the reference/directing video

Let Me Explain:

1. Rehearse the song!
   - Rehearse with the reference video so you know what to expect. The better you know the song, the better you will sound, and the easier it will be to follow the instructions on the video.
   - Access Reference Track Videos on the *United Voices* website: www.unitedvoices.ucsf.edu
2. Record a video of you singing with the reference/directing video
   You will be recording yourself singing on your phone, while watching the reference video on your tablet/computer and listening to it through your headphones.

SET UP:

- Find a **quiet** place to record. Make sure it is **well lit** (face a window, set up a lamp in front of you, etc). Try to avoid filming with a bright window behind you.
- Fix your background! A light-colored blank wall or plain background is best.
- Prepare your phone to record video
- Prepare your tablet/computer to play the reference video
- Set up your devices:
  - Prop up your PHONE (use a stack of books, boxes, a chair on a table, etc.) so that it is approximately 2 feet away from you at FACE LEVEL so that you're looking either straight ahead or even *slightly* upwards into your phone's camera.
  - Set up your TABLET/COMPUTER near or next to your phone, or behind you so you can see the Music Director's directing video and look at the camera without turning your head.

FRAMING:

- Make sure your phone is in HORIZONTAL orientation

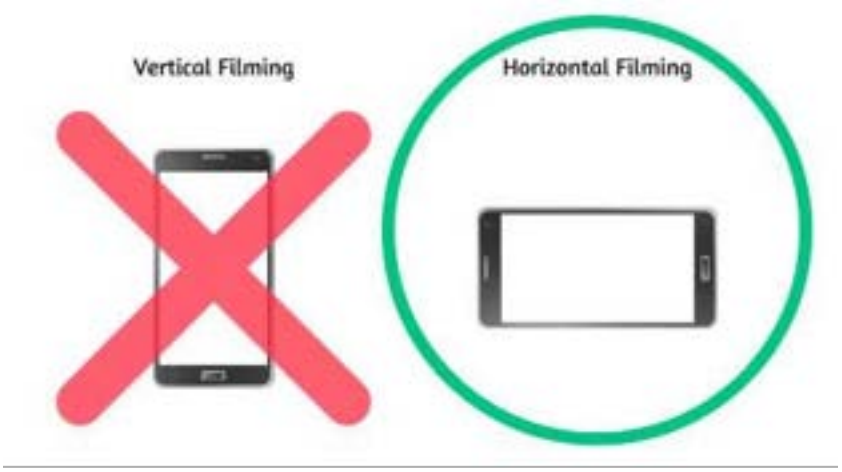

- The camera should be FACE LEVEL about 2 feet away from you.
- Don't give yourself too much or too little headroom (use the grid as a visual reference).

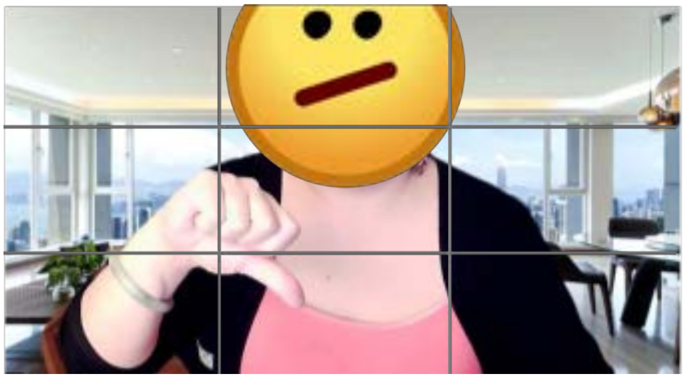

Too little headroom, the top of the head has been cut off!

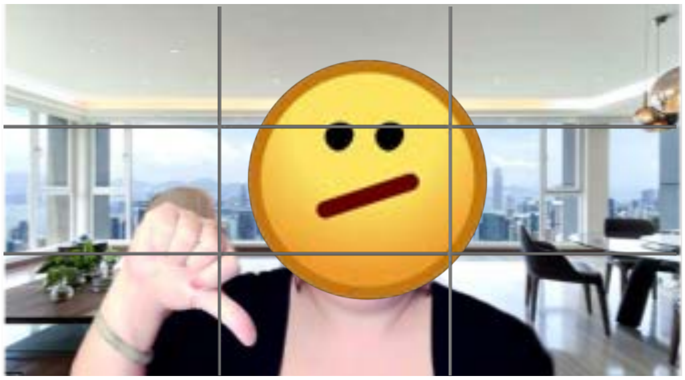

Too much headroom, face should NOT be centered.

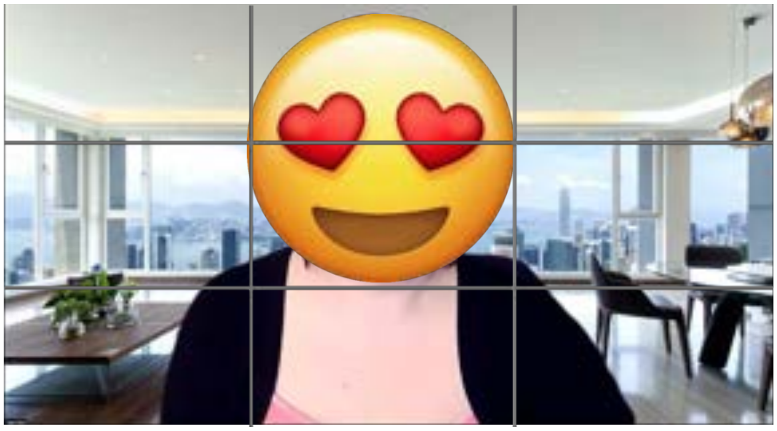

Juuuuuuust right! There still just a bit of room at the top of the head, and the chin should not pass the lower line.

TIME TO RECORD!

- Connect your headphones to your TABLET/COMPUTER
- Clean your PHONE camera lens (seriously, just do it)
- Hit record on your PHONE FIRST, then play the reference video
- Follow the instructions in the video and SING IT OUT!
  - DO NOT start and stop the video recording
  - If you mess up, you'll have to start all over again (which is why rehearsing will save you a lot of time!)
  - Keep your face in relatively the same place while you're rocking and emoting.
  - Look at the camera as much as you can (knowing the song well, can also help with this!)
- Count 5 seconds in your head before you move to turn off the video. Remember to smile!
- YOU DID IT, you recorded your video! If you recorded it correctly, it should be a video of you singing the song acapella.

3. Submit your video in ONE of two ways:
   - Go to https://www.transfernow.net/en/
     - Press "Start" to select file from your phone
     - Enter [redacted] into "Your contact's email address"
     - In the subject AND message fields please enter:

       VoicePart-FirstName LastName (e.g., Bass-Paul Daniels)

     - It will take anywhere from 10 minutes to an hour to upload depending on your connection.
     - You should receive a confirmation email directly from TransferNow letting you know that your file was transferred successfully.
     - Watch the tutorial for android here: [redacted]
     - Watch the tutorial for iPhone here: [redacted]
     - TIPS for a faster transfer (hopefully):
       - Turn on wifi on your device so you don't use up your data
       - Put your device close to the router when you're transferring
       - Once you start your upload, don't do anything else on your phone (take a nap, do the dishes, read a book)

– OR –

- Upload your video to your personal cloud storage service (Google Drive, Dropbox, iCloud, etc.) and email a downloadable link to [redacted] with the subject line:

  VoicePart-FirstName LastName (e.g., Bass-Paul Daniels)

Please **DO NOT** send your video via text/iMessage/whatsApp, etc. It degrades the quality of the video and we want to see you in your full HD glory!

YOU DID IT!
Thank you for participating!
