# Supplementary material for: United Voices Group-Singing Intervention to Address Loneliness and Social Isolation Among Older People With HIV During the COVID-19 Pandemic: Intervention Adaption Study
Source: JMIR Form Res. 2024 Oct 8;8:e60387. doi: 10.2196/60387 (PMC11496909; doi:10.2196/60387)
Supplement: Multimedia Appendix 3 [file formative_v8i1e60387_app3.docx]

**Multimedia Appendix 3.** Topic expert recommendations and adaptations for COVID-19 and cultural relevance for older people living with HIV.

| Core elements | Outcomes | Topic expert recommendations | Adaptations for virtual delivery during the COVID-19 pandemic | Adaptations for cultural relevance |
| --- | --- | --- | --- | --- |
| Engage with the conductor, fellow singers, and perform musical selections | - Focused attention and executive function | - Innovate engagement, feedback, and troubleshooting problems with choir directors and choir members | - Convene choir rehearsals and public performances on Zoom (Zoom Video Communications) - Have choir members self-record songs and send tracks to music producers, who then assemble recordings | - Music directors select songs with themes and messages that are uplifting and encouraging and connect to long-term HIV/AIDS survivorship |
| Learn and recall new music (lyrics, melody, pitch, and rhythm) | - Stimulate verbal learning and memory | - Notify participants about song repertoire upon orientation - Find ways to facilitate practicing songs independently in preparation for rehearsals | - Email syllabus of songs to choir members to assist with preparation and memorization before rehearsal - Email song tracks to choir members so that they can listen and replay songs as they are learning | - Music directors select songs with themes and messages that are uplifting and encouraging and connect to long-term HIV/AIDS survivorship |
| Singing | - Reduce depressive symptoms and anxiety and increase positive emotions through breathing and creating music | - Have music directors take on a leadership role in communicating with choir members about songs and building self-efficacy in singing their parts through rapport | - Designate a portion of the Zoom rehearsal for vocal warm-ups and practicing songs - Ensure that choir members have 2 mobile devices: one for listening to a song track and another for recording their singing - Establish regular office hours and drop-in “Help Desk” on Zoom and in person for technology-related questions | - Music directors select songs with themes and messages that are uplifting and encouraging and connect to long-term HIV/AIDS survivorship |
| Something to do and regular activity | - Increased sense of interest in daily life and belonging due to being part of something | - Retain the weekly frequency of choir rehearsals as implemented in the original intervention - Communicate responsibilities and expectations for choir participation over the project period | - Outline attendance policy and ground rules and expectations for participation in the virtual program in a choir syllabus - Ground rules include being present, avoiding distractions, and having their camera on during rehearsals | - None |
| Build social network and make new friends | - Increase social support, decrease feelings of loneliness, and develop group identity and pride | - Emphasize the religious-neutral and secular orientation of the choir to reduce anticipated stigma and promote social cohesion - Recruit choir members from diverse organizations within the community that are not solely faith based - Designate choir rehearsal time or external time for socialization | - Lead a 30-minute structured socialization segment during each weekly rehearsal | - Hire a project recruiter to ensure that outreach and advertisements are inclusive and reach groups that are in and outside of faith-based settings - Select songs that have meaning and context outside of religious affiliation - Select songs that foster community connectedness over life events, relevancy, feelings, and emotions evoked by the lyrics - Have principal investigator lead socialization break discussions about current life events, longtime HIV/AIDS survivorship, and social isolation (due to HIV or COVID-19) - Music directors discuss the songs and prompt choir members to process and discuss what the songs mean to them and their personal lives |
